# Characterization of a New Chitosanase from a Marine *Bacillus* sp. and the Anti-Oxidant Activity of Its Hydrolysate

**DOI:** 10.3390/md18020126

**Published:** 2020-02-19

**Authors:** Chunrui Ma, Xiao Li, Kun Yang, Shangyong Li

**Affiliations:** College of Basic Medicine, Qingdao University, Qingdao 266071, China; 15314203317@163.com (C.M.); lilix0823@163.com (X.L.); ya997720478@163.com (K.Y.)

**Keywords:** chitosanase, pH-stability, *Bacillus* sp. Q1098, chitooligosaccharide, anti-oxidant activity

## Abstract

Chitooligosaccharide (COS) has been recognized to exhibit efficient anti-oxidant activity. Enzymatic hydrolysis using chitosanases can retain all the amino and hydroxyl groups of chitosan, which are necessary for its activity. In this study, a new chitosanase encoding gene, *csnQ*, was cloned from the marine *Bacillus* sp. Q1098 and expressed in *Escherichia coli*. The recombinant chitosanase, CsnQ, showed maximal activity at pH 5.31 and 60 °C. Determination of CsnQ pH-stability showed that CsnQ could retain more than 50% of its activity over a wide pH, from 3.60 to 9.80. CsnQ is an endo-type chitosanase, yielding chitodisaccharide as the main product. Additionally, in vitro and in vivo analyses indicated that chitodisaccharide possesses much more effective anti-oxidant activity than glucosamine and low molecular weight chitosan (LMW-CS) (~5 kDa). Notably, to our knowledge, this is the first evidence that chitodisaccharide is the minimal COS fragment required for free radical scavenging.

## 1. Introduction

Chitin, an insoluble linear poly-acetylaminosaccharide made up of β-1,4-linked N-acetyl-D-glucosamine (GlcNAc), is the second most abundant natural biomass after cellulose on earth [1]. As its most important derivative, chitosan is a biopolymer obtained by partial or complete deacetylation of chitin, and consists of GlcNAc and D-glucosamine (GlcN) residues [2]. Chitosan has immense potential for application in multiple fields owing to its unusual physicochemical properties such as biocompatibility, biodegradability and low toxicity [1]. Currently, chitosan is widely utilized in biomedical applications, as an absorbable surgical suture, as a component of artificial skin, in wound healing accelerators, and in immunoregulatory agents [3].

Chitooligosaccharide (COS), is water-soluble, less viscous and low molecular weight depolymerized derivative of chitosan, and is commonly superior to chitosan polymers in several aspects [1]. Recently, COS has been a subject of considerable attention in terms of its role in many biological applications, which correlates well with its antimicrobial [4], anti-inflammatory [5], anti-oxidant [6], anti-photoaging [7], anti-tumor [8] activities as well as immuno-enhancer effects [9]. The chemical structures and molecular sizes of COS are primary factors that significantly affect the biological activities [10]. Mendis et al. proposed that COS with molecular weight (MW) < 1 kDa alleviated intracellular free radical damage in cellular oxidation systems in an in vitro set up, and also demonstrated that treatment with COS resulted in attenuation of the promoter activity of the NF-κB gene [11]. Artan et al. suggested that sulfated COS (MW of 3–5 kDa) is the most effective compound in stopping the replication of HIV-1 virus [12]. The anti-obesity effect of COS could be attributed to its property of suppression of adipocyte differentiation. Cho et al. demonstrated that COS with MW of 1–3 kDa is most efficient in inhibiting adipocyte differentiation [13]. Lee et al. showed that COS with MW of 10 kDa possesses a higher anti-microbial efficacy against *V. vulnificus* than that with 1 kDa MW in vitro and in vivo [14]. Interestingly, Fernandes et al. also proved that lower molecular weight COS exerts a stronger anti-microbial effect towards Gram-negative bacteria, and shows an opposite effect towards Gram-positive bacteria [15].

Chitosanase (EC.3.2.1.132), a type of glycoside hydrolase (GH), can catalyze hydrolysis of the β-1,4-linked glycosidic bond in chitosan [16]. Enzymatic methods employing chitosanases are favorable for degrading chitosan into COS. Compared with chemical and physical methods, enzymatic hydrolysis has many advantages, such as (i) specific degradation without distorting the amino and carboxyl groups structure of chitosan, which is necessary for its activity; (ii) environmental-friendliness of the process that does not consume quantities of acidic reagents; and (iii) easy control of reaction products under mild conditions [17]. To date, various chitosanases have been isolated from different organisms, including fungi, plants, and bacterium. In the Carbohydrate-Active Enzymes (CAZy) database, chitosanases have been classified as GH families 5, 7, 8, 46, 75, and 80 [18]. Enzymes with special properties are favored in industrial production. Since the degree of polymerization (DP) directly affects the biological activity of COS, it is essential to find a chitosanase with a single reaction product for structural and functional research. However, the reaction products of most reported chitosanases are a mixture of DP2-DP6, which are difficult to separate. In our previous study, we have designed and synthesized an affinity resin to screen and isolate native chitosanases directly from bacterial culture supernatants [19]. Using this protocol, a chitosanase, CsnQ, was isolated and purified from the marine bacterium *Bacillus* sp. Q1098 isolated from the sediment of Yellow Sea.

In this study, we cloned a new encoding gene, *csnQ*, from the marine *Bacillus* sp. Q1098 and expressed it in *Escherichia coli* BL21 (DE3). To further assay the potential applications of this chitosanase, the biochemical properties of CsnQ were determined using the purified enzyme. Afterwards, anti-oxidant activity was determined using the reaction products of CsnQ. The result indicated that CsnQ is a potential candidate for biotechnological application in the future.

## 2. Results and Discussion

### 2.1. Sequence Analysis of CsnQ

In this study, the putative chitosanase gene, *csnQ*, obtained from the marine isolate *Bacillus* sp. Q1098, contained an intact open reading frame (ORF) of 861 bp and encoded a protein, CsnQ, consisting of 287 amino acid residues. Based on results of signal peptide analysis, CsnQ contained a putative signal peptide (Met^1^ to Gly^22^) in its N-terminus. In addition, the theoretically isoelectric point (pI) and MW of CsnQ were 5.89 and 30.6 kDa, respectively. Moreover, based on the National Center for Biotechnology Information (NCBI) conservative domain database (CDD) search, CsnQ was found to be a putative chitosanase with its conservative domain belonging to family GH46. NCBI blast results suggested that CsnQ shared high sequence similarity with several family GH46 chitosanases from *Amycolatopsis* sp. CsO-2 [GenBank: BAA94840] (82.98%), *Kitasatospora setae* KM-6054 [GenBank: BAJ27342] (82.48%) and *Kitasatospora albolonga* ATCC 27414 [GenBank: AYH64862] (78.63%).

**Figure 1:**
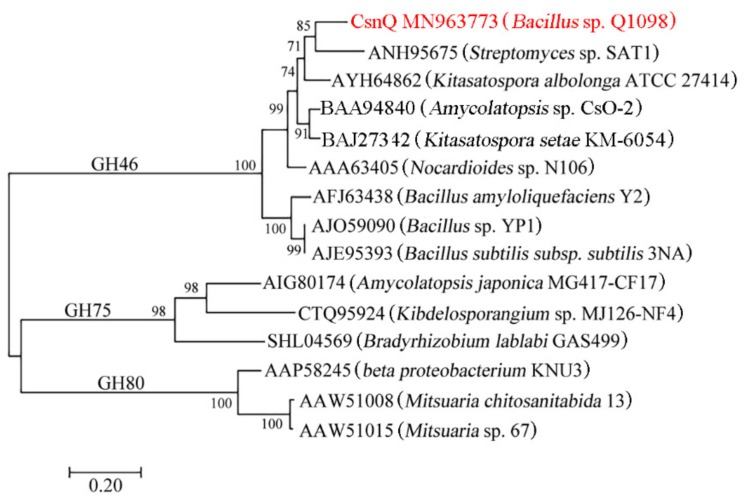
Phylogenetic analysis of CsnQ and several other reported chitosanases. Branch-related numbers are bootstrap values (confidence limits) representing the substitution frequency of each amino acid residue.

**Figure 2:**
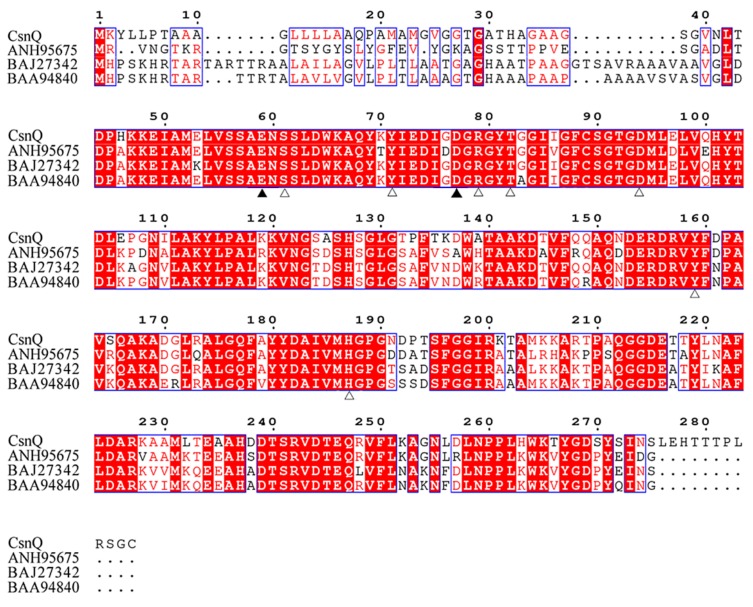
Sequence comparison of CsnQ with other chitosanases from GH 46 family. The BLAST 2.0 program with the function Sequence similarity searches was used. Identical amino acid residues are boxed in red, and amino acid residues above a 70% consensus are boxed in a pale pane. The hollow triangle marks the sugar-binding site. Solid triangle marks catalytic sites.

As shown in Figure 1, a phylogenetic tree was constructed, which contained CsnQ and other reported chitosanases from families GH46, GH75 and GH80. As expected, CsnQ and other GH46 family chitosanases from *Streptomyces* sp. SAT1 [GenBank: ANH95675] and *Nocardioides* sp. N106 [GenBank: AAA63405], formed a bigger cluster, whereas the families GH75 and GH80 members formed other two clusters. As shown by multiple sequence alignment (Figure 2), the conserved region labeled by triangles in CsnQ was related to the substrate binding and catalytic activities of the family GH46 [20,21,22,23,24]. These results indicated that CsnQ was a new member of the family GH46.

### 2.2. Expression, Purification and Characterization of CsnQ

**Figure 3:**
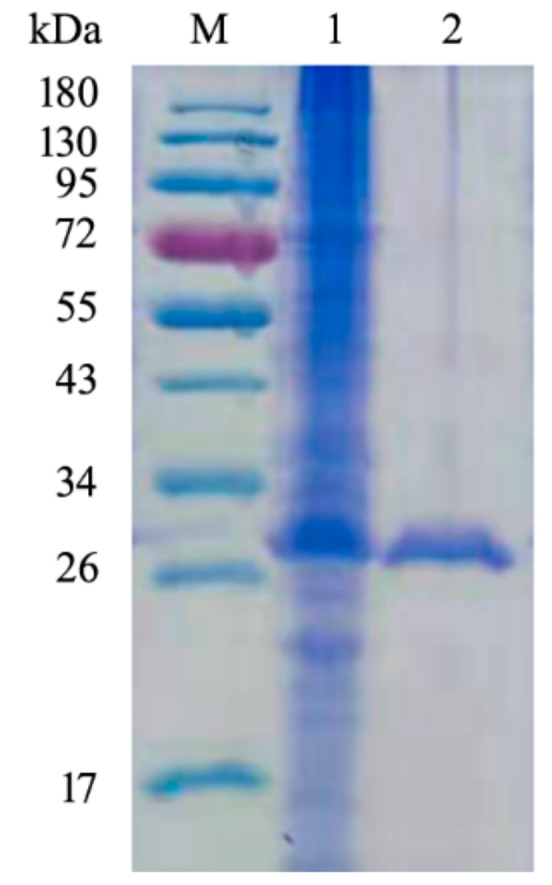
SDS-PAGE analysis of the recombinant CsnQ. Lane M, protein marker; Lane 1, the crude CsnQ; Lane 2, the purified CsnQ.

The constructed expression strain *E. coli* BL21-pET22b-*csnQ* was incubated in Luria Bertani (LB) broth and recombinant CsnQ was purified by (histidine)_6_-Ni^2+^-nitrilotriacetate (Ni-NTA) affinity column. The specific activity of purified CsnQ was 371.6 U/mg. Purified CsnQ was also analyzed by sodium dodecyl sulfate polyacrylamide gel electrophoresis (SDS-PAGE). As shown in Figure 3, a single band was observed on the gel. The MW of purified CsnQ was found to be approximately 30 kDa, which corresponded well to the expected MW of the theoretical protein (30.6 kDa).

**Figure 4:**
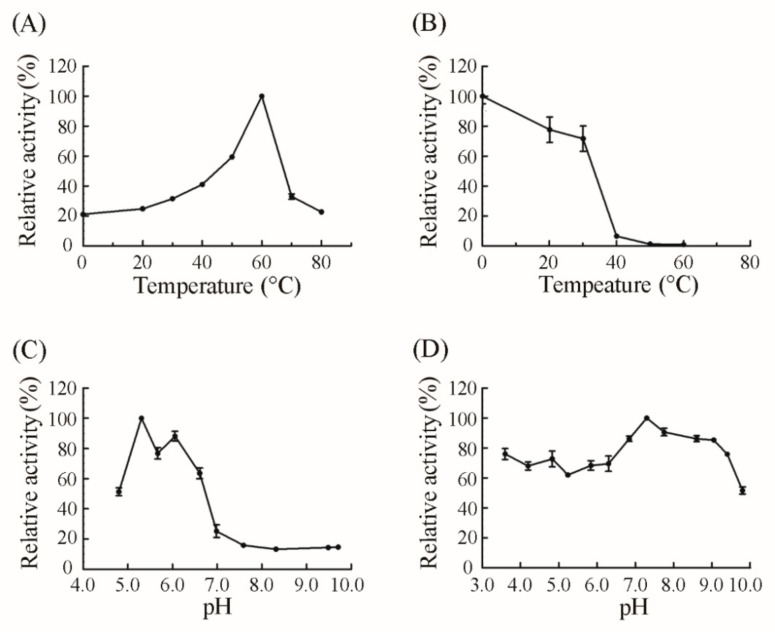
Characterization of CsnQ. (**A**) The optimal temperature of CsnQ; (**B**) The thermal-stability of CsnQ. Taking the activity of the pre-incubated CsnQ at 0 °C as 100%, the residual activity at the optimal temperature of 60 °C was detected; (**C**) Optimal pH for the relative activity of CsnQ was determined in the assay (pH 4.80–9.71) containing equal volumes of Britton-Robinson buffers (pH 3.60–9.80) and substrate solution [containing 0.3% (*w*/*v*) soluble chitosan, pH 6.0] at the optimal temperature; (**D**) The pH stability of CsnQ was analyzed by measuring the residual activity at the optimal temperature and pH after the enzyme was pretreated at +4 °C for 12 h in different pH of Britton-Robinson buffers (pH 3.60–9.80). The mean values ± standard deviations of three experiments each in 3 replicates are shown.

The biochemical properties of CsnQ were then analyzed using the purified enzyme. The optimal reaction temperature was found to be 60 °C (Figure 4A), which was higher than that of other chitosanases, such as CsnM from *Pseudoalteromonas* sp. SY39 (40 °C) [25], CsnB from *Bacillus* sp. BY01 (35 °C) [26], Csn-PD from *Paenibacillus dendritiformis* (45 °C) [27], CSN from *Penicillium* sp. D-1 (48 °C) [28], CSN-SP from *Bacillus* sp. DAU101 (50 °C) [29] and multiple untitled chitosanases from *Bacillus* sp. strain CK4 (55 °C) [30] and *Janthinobacterium* sp. strain 4239 (45 °C) [16] (Table 1). Meanwhile, CsnQ could retain 77.72% and 71.79% of the original activity after 60 min of incubation at 20 and 30 °C, respectively. The residual activity of purified CsnQ reduced dramatically after 60 min of incubation at temperatures above 30 °C (Figure 4B). Additionally, in Britton-Robinson buffers (pH ranging from 4.80–9.71), chitosanases from *Paenibacillus dendritiformis*, *Bacillus* sp. DAU101 and *Bacillus* sp. strain CK4 have the highest activity at pH 7.0, 7.5 and 7.5, respectively [27,29,30] (Table 1), while the optimum pH of purified CsnQ was 5.31 (Figure 4C). It is difficult to dissolve chitosan in neutral and alkaline conditions and it requires an acidic environment. Therefore, CsnQ was considered as a more appropriate enzyme catalyst in the hydrolysis of chitosan than other chitosanases due to its optimal activity in acidic conditions. CsnQ was also found to be quite pH-tolerant, and could maintain over 50% of its original activity after 12 h of incubation at +4 °C at a wide range of pH (3.60–9.80). Remarkably, it could also retain relatively high activity (>85%) after incubation at pH ranging from 6.84 to 9.05 (Figure 4D). Therefore, wide pH stability and high activity in acidic conditions make CsnQ a promising candidate for chitosan degradation.

The effect of metal ions and reagents (concentration of 5 mM) on the activity of CsnQ is shown in Supplementary Table S1. The activity of CsnQ was enhanced by K^+^, Na^+^, Mg^2+^, Li^+^, NH_4_^+^, Fe^2+^, Cu^2+^, Ba^2+^, Co^2+^, Ca^2+^ and Zn^2+^ among the tested metal ions. The metal ion Fe^3+^ slightly inhibited the activity of CsnQ while inhibition by Al^3+^ was obvious. Furthermore, chemical reagents such as Triton × 100, Tween, SDS and EDTA, significantly inactivated CsnQ.

### 2.3. Action Mode and Reaction Product Analysis

**Figure 5:**
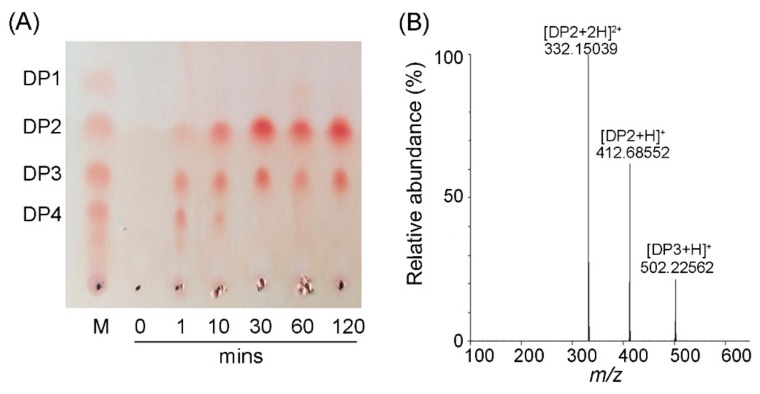
Analysis of CsnQ reaction products. (**A**) TLC analysis of the degradation products of chitosan by CsnQ. The reaction system contained 450 μL 0.3% (*w*/*v*) chitosan substrate (pH 6.0) and 50 μL purified CsnQ at 60 °C, and the reaction mixture were analyzed at the indicated times. Lane M: standard monomer (DP1) and chitosan oligomers (DP2–4); Lanes 0–120: enzymatic hydrolysates of chitosan incubated at 60 °C for 0, 1, 10, 30, 60 and 120 min, respectively. (**B**) ESI-MS analysis of end products derived from hydrolysis of chitosan by CsnQ.

The action mode of CsnQ and its reaction product were analyzed by thin layer chromatography (TLC) (Figure 5A). In general, endo-type chitosanases hydrolyze chitosan into a mixture of COS, while exo-type chitosanases hydrolyze chitosan polymers and COS into monomers (DP1) [25]. Time course analysis indicated that CsnQ was an endo-type enzyme owing to its rapid rate of depolymerization, rise in polydispersity, and production of various oligomers. As shown in Figure 5A, the chitosan polymer was rapidly degraded after a reaction time of only 1 min. The degradation products at 10 min were a mixture of DP2, DP3 and DP4. After 30 min of reaction, two clear spots were observed on the TLC plate, and the migration rate was in agreement with chitodisaccharide and chitotrisaccharide markers, indicating that the final degradation products of CsnQ were DP2 and DP3.

Positive-ion electrospray ionization mass spectrometry (ESI-MS) was further used for analyzing the final reaction products of CsnQ (Figure 5B). The main spectra observed were 332.15 *m*/*z* [DP2+2H]^+^ and 412.69 *m*/*z* [DP2+H]^+^, which corresponded to the molecular mass of chitodisaccharide. The spectrum observed at 502.23 *m*/*z* [DP2+H]^+^ corresponded to the molecular mass of chitotrisaccharide. These results indicated that the main reaction product of CsnQ is chitodisaccharide. Various endo-type chitosanases have been cloned and characterized so far. The reaction products of these chitosanases are mainly a mixture of DP2-DP6 such as those of chitosanases from *Paenibacillus dendritiformis* [27], *Bacillus* sp. DAU101 [29] and *Bacillus* sp. strain CK4 [30] (Table 1). The end products of chitosanase from *B. cereus* TKU034 are a mixture of DP3-DP9, which are obviously hard to separate [31]. Hence, CsnQ is an enzyme with well-distributed products having tremendous potential in industrial production.

### 2.4. Anti-Oxidant Activity of COS

**Figure 6:**
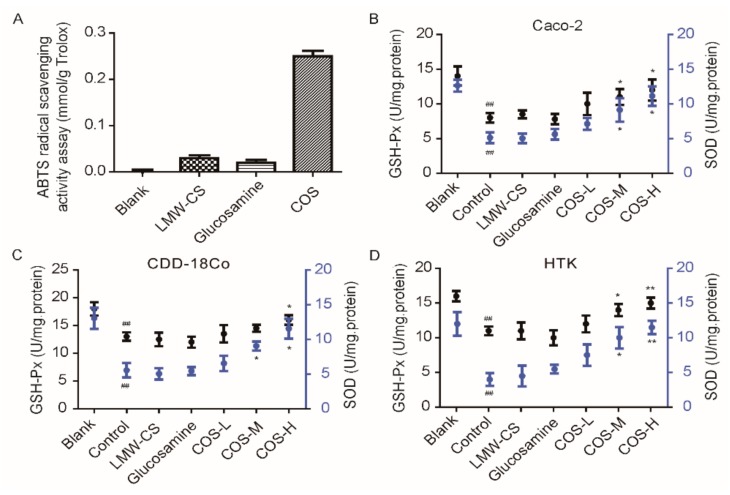
Anti-oxidant activity of COS. (**A**) In vitro analysis of CsnQ anti-oxidant activity using ABTS method, the contents of GSH-Px and T-SOD were measured in (**B**) Caco-2, (**C**) CDD-18Co and (**D**) HTK cell types. All the results were expressed as mean values ± S.E. obtained from three separated experiments. Blank, no treatment; control, H_2_O_2_ treated group. COS-L, 100 μg/mL; COS-M, 300 μg/mL and COS-H, 500 μg/mL. All of the drugs (COS-L, COS-M, COS-H, LMW-CS, and Glucosamine) were dissolved in 50 mM Tris-HCl buffer (pH 8.0). ## *p* < 0.01 indicated a highly significant difference from the blank group. * *p* < 0.05 and ** *p* <0.01 indicated significant difference from the H_2_O_2_-treated group (the control group).

Recently, several reports have shown that COS can be potentially used as anti-oxidants, owing to its free radical scavenging ability via hydrogen transmission mechanism [32]. The amino and hydroxyl groups of COS can react with unstable free radicals to form stable macromolecular free radicals. Therefore, the free radical scavenging efficacy of COS directly correlates with its structural properties [33]. Previous studies have indicated that the anti-oxidant activities of COS are mainly influenced by its DP and MW, and this ability increases with decreasing MW [1]. Compared with acid hydrolysis, enzymatic hydrolysis reaction using chitosanases could be conducted under mild conditions and is favored for well-defined COS, with the molecular size of the products well-distribution [34]. In this study, we verified by 2,2′-azino-bis(3-ethylbenzothiazoline-6-sulfonate) (ABTS) method that the reaction products of CsnQ in vitro have better radical scavenging efficacy (0.25 mmol/g) than LMW-CS (~5 kDa) (Figure 6A). The end product of CsnQ hydrolysis was mainly chitodisaccharide, and glucosamine on its own did not have anti-oxidant capacity (0.03 mmol/g), indicating that chitodisaccharide is the minimal fragment for anti-oxidant activity. In TLC analysis (Figure 5A), DP1 was not be detected in the products of chitosan degradation by CsnQ. Hence, using CsnQ to degrade chitosan is of great advantage in anti-oxidant therapy.

In general, cells respond to oxidative stress by making adaptive changes to prevent cellular damage and improving survival [35]. Recently, several studies verified that COS has potent anti-oxidant activities. A recent study investigated COS for its anti-oxidant and anti-inflammatory capabilities in human embryonic hepatocytes (L02 cells), which has crucially high 1,1-diphenyl-2-picrylhydrazyl (DPPH) radical scavenging activity and protects against hydrogen peroxide (H_2_O_2_) induced DNA damage [36]. In another study, COS was confirmed for its hepatoprotective effect against tert-butylhydroperoxide (t-BHP)-induced toxicity employed in Chang liver cells [37] (Table 2). During normal metabolism, our human body produces a certain number of oxidative free radicals. Total-superoxide dismutase (T-SOD) and glutathione peroxidase (GSH-Px) have a role in removing these oxidation free radicals to balance metabolism. T-SOD and GSH-Px were commonly used for biomarkers in anti-oxidant analysis. In this study, the effects of chitodisaccharide on the protective activity of T-SOD and GSH-Px are shown in Figure 6B–D. The results indicated that after the Caco-2, normal human colon fibroblast cell line (CDD-18Co) and human corneal stromal cells (HTK) were all incubated with H_2_O_2_ (as the control group), the amount of T-SOD and GSH-Px released from cells were significantly decreased. However, when COS was added at concentrations of 100, 300, and 500 μg/mL, the production of T-SOD and GSH-Px were significantly higher than that in control group, but not in LMW-CS and glucosamine. These results indicated that COS might protect cells from oxidative-damage by the production of T-SOD and GSH-Px in a concentration-dependent manner.

## 3. Materials and Methods

### 3.1. Strains and Materials

The marine bacterium *Bacillus* sp. Q1098 was isolated from the sediment of Yellow Sea in China. The *E. coli* strains DH5 and BL21 (DE3) (Solarbio, Beijing, China) were grown in Luria Bertani (LB) medium, which was used for plasmid construction and gene expression host. Two bacterial strains were cultured in LB broth including ampicillin (50 μg/mL) at 37 °C. The expression vector pET22b (+) was purchased from Takara (Dalian, China). Chitosan (degree of deacetylation ≥ 95%, viscosity: 100–200 mpa.s) was purchased from Aladdin Biochemical Technology Co., Ltd. (Shanghai, China).

### 3.2. Sequence Analyses

The draft genome of *Bacillus* sp. Q1098 was determined using a second-generation sequencer in our lab. A putative GH family 46 gene, *csnQ*, was identified and its nucleotide sequence was deposited in the GenBank database (accession number: MN963773). In addition, the gene was selected for gene cloning and chitosanase expression. The complete open reading frame (ORF) of CsnQ was discovered using the program ORF Finder, additionally, the signal peptide of CsnQ was analyzed using the detaibio Signal Peptide Analysis tool in detaibio Afterwards, the conserved domain of CsnQ was analyzed through comparison with the CDD in the NCBI database. Moreover, the pI/MW tool in Expasy was applied for calculating and analyzing the theoretically pI and the MW of CsnQ. Additionally, the NCBI Basic Local Alignment Search Tool (BLAST) algorithm was utilized to search for protein sequences similar to CsnQ. EsPript 3.x was used for multiple sequence alignment. Moreover, the phylogenetic trees of CsnQ and other reported chitosanases were established in mega7.0 by the bootstrapping neighbor-joining method.

### 3.3. Heterologous Expression of Recombinant CsnQ

The gene expression primers are *csnQ*-EF (CATGCCATGGATGAAATACTTATTACCAAC) and *csnQ*-ER (CCGCTCGAGACAACCAGAACGTAATGG). Two primers possessed the restriction endonuclease cleavage site and protective base of *Nco* I and *Xho* I at the 5′ ends, respectively. The PCR product and the expression vector pET22b (+) was digested with *Nco* I and *Xho* I restriction endonuclease. Then, the digested PCR product was ligated into the vector. Furthermore, the recombinant plasmid, pET22b (+)-*csnQ*, was transformed into *E. coli* BL21 (DE3) for gene expression.

### 3.4. Purification of Recombinant CsnQ 

The *E. coli* BL21 (DE3)-pET22b (+)-*csnQ* strain was cultured in LB broth and induced at OD600 of 0.6 with 0.1 mM isopropyl β-D-1-thiogalactoside (IPTG) at 20 °C and shaking at 200 rpm. After incubation for 16 h, the recombinant strains were harvested by centrifugation (12,000 rpm) at 4 °C for 10 min. The supernatant (100 mL) was loaded onto a Ni-NTA column (GE Healthcare, Little Chalfont, Buckinghamshire, UK) in the AKTA150 automatic intelligent protein purification system (GE Healthcare, Little Chalfont, Buckinghamshire, UK). The target enzyme was eluted by elution buffer which contained 500 mM NaCl and 100 mM imidazole in 50 mM phosphate buffer (pH 7.6). The MW of CsnQ was analyzed by SDS-PAGE, followed by staining the protein band using the coomassie brilliant blue. In addition, we utilized a bicinchoninic acid (BCA) protein assay kit (Beyotime Biotechnology, Shanghai, China) to measure the concentration of purified CsnQ with bovine serum albumin (BSA) as a standard.

### 3.5. CsnQ Activity Assay

CsnQ activity was determined by the 3,5-dinitrosalicylic acid (DNS) method. The reaction mixture was composed of 50 μL purified enzyme and 450 μL substrate solution [containing 0.3% (w/v) soluble chitosan, pH 6.0]. After 10 min of reaction at optimal temperature (60 °C), 375 μL DNS was added to terminate the reaction [25]. Thereafter, the mixture was boiled for 10 min and cooled to room temperature (25 °C), and centrifuged at 12,000 rpm for 5 min to remove the sediment subsequently. Then, the content of reducing sugar produced in the supernatant was measured at 520 nm. The definition of one unit of chitosanase activity is the amount of enzyme requested for releasing 1 µmol D-glucosamine-equivalent reducing sugar per minute under the above assay conditions. A standard curve with known concentrations of D-glucosamine was created.

### 3.6. Effect of Temperature, pH, Metal Ions and Chemical Compounds on CsnQ Activity

The optimal temperature for CsnQ activity was confirmed through measuring the changes in CsnQ activity from 0 to 80 °C. To determine its thermo-stability, the residual enzyme activity after 60 min of incubation at various temperatures (0–60 °C) was determined. On the other hand, the optimal pH of CsnQ was determined in the assay (pH 4.80–9.71), which contained equal volumes of Britton-Robinson buffers (pH 3.60–9.80) and substrate solution [containing 0.3% (*w*/*v*) soluble chitosan, pH 6.0]. Furthermore, the pH stability of CsnQ was analyzed through determining its residual activities after 12 h of pretreatment at 4 °C in different pH conditions of Britton-Robinson buffers (pH 3.60–9.80) at a ratio of 1:9. To examine the impacts of chemical compounds and metal ions on the enzyme activity, a variety of mixture and different metal ions were added into the reaction system at a 5 mM final concentration. At the same time, the reaction mixture without metal ions or chemical compounds was used as the controls. The results of the reaction were expressed as the mean value ± s.d. of triplicate experiments.

### 3.7. Analysis of the CsnQ Reaction Products

The final degradation products were detected by TLC approach using ninhydrin stain method, wherein a reaction system containing 450 μL 0.3% (*w*/*v*) chitosan substrate (pH 6.0) and 50 μL purified CsnQ, and the samples were detected at 60 °C for 1, 10, 30, 60 and 120 min, respectively. Afterwards, the reaction products were analyzed by the TLC plate, while the developing solvent is ammonia/water/isopropanol (3:27:70, *v*/*v*). Then, the presence of sugars on the plates was visualized after spraying detection chemical (0.5% ninhydrin dissolved in ethanol) and warming at 80 °C for 30 min. Furthermore, the composition of the degradation products was further analyzed through ESI-MS.

### 3.8. In Vitro Analysis of the Anti-Oxidant Activity of COS

The reaction product of CsnQ was lyophilized by freeze dryer. Then, the COS powder was dissolved in 50 mM Tris-HCl buffer (pH 8.0) for anti-oxidant determination. In vitro anti-oxidant activity was analyzed by the Total Anti-oxidant Capacity Assay Kit with ABTS method (ABTS method, Beyotime Institute of Biotechnology, Shanghai, China). This method measures the oxidation under appropriate agents of ABTS in ABTS+, which is a blue-green chromophore with a characteristic absorption at 734 nm. The results were shown as equivalent concentration of 2 mmol/g Trolox (a water-soluble analogue of vitamin E) in order to be standardized.

### 3.9. Cell Culture and Treatment

All the cell lines were purchased from American Type Culture Collection (Manassa, VA, USA). Cell culture, subculture and cryopreservation were carried out according to product manual. 2.0 mL of cells (5 × 10^3^ cells/well) were added into a 6-well plate and incubated for 12 h. After discarding the culture medium, a total of 2 mL of H_2_O_2_ (100 nM/mL) was added to each well except to the blank and incubated for further 24 h. Then, fresh Dulbecco’s modified Eagle’s medium (DMEM) was added to all wells. The volumes of LMW-CS (500 μg/mL), glucosamine (500 μg/mL), COS of concentration at 100 μg/mL (COS-L), COS of concentration at 300 μg/mL (COS-M), COS of concentration at 500 μg/mL (COS-H) were added to the respective wells and cultured for 24 h. After oxidative damage, cells were treated with or without LMW-CS, glucosamine and COS. Following the products instruction, the content of T-SOD and GSH-Px in the supernatant was measured using the specific determination kits (Dakewe Biotech Co., Ltd., Shenzhen, China).

### 3.10. GSH-Px Activity

GSH-Px activity was estimated utilizing a commercial kit provided by Dakewe and was expressed as mU/mg protein. This enzymatic reaction was triggerd with the addition of cumene hydroperoxide, in which the Nicotinamide Adenine Dinucleotide 2′-Phosphate reduced tetrasodium salt (NADPH), reduced GSH and GSH reductase was involved. The GSH-Px level was detected at a wavelength of 340 nm using a spectrophotometer.

### 3.11. T-SOD Activity

T-SOD activity in cell lysates was measured utilizing a commercial kit provided by Dakewe. The method is based on xanthine and xanthine oxidase generating superoxide anion, a substance which is able to react with tetrazolium chloride followed by forming a yellow color formazan dye. T-SOD level was detected at a wavelength of 450 nm and the enzyme activity was given as U/mg protein.

## 4. Conclusions

In summary, the encoding gene, *csnQ*, was cloned and expressed in *Escherichia coli*, and recombinant CsnQ was purified and characterized. CsnQ can serve as an excellent candidate for industrial applications owing to its special properties, such as low optimal pH, wide pH-stability and single reaction product. Moreover, our future analysis will focus on its three-dimensional structure and reaction mechanisms. Notably, we have found that the reaction product of recombinant CsnQ is mainly chitodisaccharide (DP2), and we also demonstrated that DP2 is the minimal fragment required for free radical scavenging activity, which greatly raises our awareness about the anti-oxidant capacity of COS.

## Figures and Tables

**Table 1:** Comparison of the CsnQ properties with other reported chitosanases.

Name	GenBank No.	Orgnism Source	Optimal pH	Stable pH Range	Optimal Temperature (°C)	Products (DP)	References
CsnQ	MN963773	*Bacillus* sp. Q1098	5.31	6.8–9.1	60	Mainly 2	This study
CsnM	MH675972	*Pseudoalteromonas* sp. SY39	5.9	5.8–7.9	40	2–3	[25]
CsnB	MN531545	*Bacillus* sp. BY01	5.0	4.6–5.8	35	2–3	[26]
Csn-PD	−	*Paenibacillus dendritiformis*	7.0	6.0–7.0	45	2–6	[27]
CSN	JF950269	*Penicillium* sp. D-1	4.0	3.0–5.0	48	−	[28]
CSN-SP	DQ316095	*Bacillus* sp. DAU101	7.5	−	50	2–6	[29]
chitosanase	AF165188	*Bacillus* sp. CK4	7.5	−	55	2–6	[30]
chitosanase	GQ487532	*Janthinobacterium* sp. 4239	5.0	−	45	1–2	[16]
chitosanase	−	*cereus* TKU034	7.0	4.5–7.5	50	3–9	[31]

**Table 2:** Comparison of the anti-oxidant properties of COS with different fragments.

Cell Types	Fragments	Concentration (μg/mL)	Anti-Oxidant Activity	References
Caco-2 CDD-18Co HTK	DP1 DP2 ~5 kDa	100, 300, 500	DP2 is the minimal fragment in free radical scavenging.	This study
Murine melanoma cell line	<1 kDa, 1–3 kDa.	10, 50, 100, 500	The effect of fragment in <1 kDa is better than in 1–3 kDa.	[11]
Human embryonic hepatocytes (L02 cells)	DP2–8	100–400	The mixture plays effective free radical scavenging capacity.	[36]
Chang liver cells	5–10 kDa, 1–5 kDa, <1 kDa.	100, 200, 500, 1000	The activity of anti-oxidant enzymes and the inhibition capacity of ROS are maximal in 5–10 kDa. The GSH (glutathione) content is highest in 1–5 kDa.	[37]
Human pancreatic β-cells	<1 kDa, 1–3 kDa, 3–5 kDa, 5–10 kDa.	500	The 3–5 kDa of COS possesses the highest anti-oxidant activity.	[38]

## Supplementary Materials

The following are available online at https://www.mdpi.com/1660-3397/18/2/126/s1, Table S1: Effects of metal ions, Triton × 100, Tween, SDS and EDTA on CsnQ activity.
