# Supplementary material for: Characterization of a New Chitosanase from a Marine Bacillus sp. and the Anti-Oxidant Activity of Its Hydrolysate
Source: Mar Drugs. 2020 Feb 19;18(2):126. doi: 10.3390/md18020126 (PMC7073567; doi:10.3390/md18020126)
Supplement: Supplementary file 1 [file marinedrugs-18-00126-s001.pdf]

Table S1. Effects of metal ions, Triton × 100, Tween, SDS and EDTA on CsnQ activity.

**Reagent Added**	**Concentration (mM)**	**Relative Activity (%)**
None	-	100 ± 0.2
Al^{3+}	5	48 ± 10.1
Fe^{3+}	5	88 ± 2.6
K^{+}	5	101 ± 4.3
Na^{+}	5	104 ± 3.9
Mg^{2+}	5	106 ± 0.7
Li^{+}	5	106 ± 3.3
NH_4^{+}	5	107 ± 3.1
Fe^{2+}	5	108 ± 2.6
Cu^{2+}	5	108 ± 3.3
Ba^{2+}	5	109 ± 4.2
Co^{2+}	5	110 ± 2.4
Ca^{2+}	5	112 ± 3.4
Zn^{2+}	5	114 ± 3.8
Mn^{2+}	5	121 ± 3.1
Triton ×100	5	5 ± 0.3
Tween	5	10 ± 1.0
SDS	5	5 ± 2.4
EDTA	5	5 ± 3.9
